# B cell activation factor (BAFF) induces inflammation in the human fallopian tube leading to tubal pregnancy

**DOI:** 10.1186/s12884-019-2324-5

**Published:** 2019-05-14

**Authors:** Jianying Xu, Xingguang Luo, Shihao Qu, Guiyan Yang, Nianchun Shen

**Affiliations:** 1 Zhuhai Municipal Maternal and Children’s Health Hospital, 543 Ningxi Road, Zhuhai, 519000 Guangdong China; 2 Division of human Genetics, Department of Psychiatry, Yale University School of Medicine, New Haven, CT USA

**Keywords:** B cell activation factor, Human fallopian tube, Salpingitis, Tubal pregnancy, Tumor necrosis factor-alpha

## Abstract

**Background:**

Tubal pregnancy is recognized as one of the most common ectopic pregnancy types. Salpingitis may result in tubal pregnancy by causing fallopian tube occlusion and hydrosalpinx. B cell activation factor (BAFF) is a proinflammatory cytokine that helps regulate both innate and adaptive immune responses. Our previous study firstly showed that BAFF immunostaining appeared on the cellular membrane and in the cytoplasm of tubal epithelial cells, and both BAFF protein and mRNA in human inflamed fallopian tubes had higher expression levels than those in normal fallopian tubes. This study aimed to elucidate the association between the expression of BAFF gene and the inflammation in the human fallopian tube leading to tubal pregnancy.

**Methods:**

We examined 70 patients undergoing salpingectomy for salpingitis (*n* = 35) and tubal pregnancy (*n* = 35). Twenty patients with benign uterine diseases undergoing complete hysterectomy and salpingectomy were recruited into control group. BAFF mRNA and protein in tissue samples were detected by qPCR and Western blotting methods. Furthermore, serum levels of BAFF, tumor necrosis factor-α (TNF-α) and interleukin (IL)-6 were measured using ELISA kits.

**Results:**

We found statistically significantly elevated expressions of BAFF mRNA or protein in whole tissue samples, and serum levels of BAFF, TNF-α and IL-6 in whole blood samples from patients with salpingitis and tubal pregnancy, in comparison to the control group.

**Conclusion:**

Based on the results, high expression of BAFF gene might induce inflammation in the human fallopian tube, suggesting its possible role in the tubal pregnancy process.

## Background

Inflammation is a response to infection, cellular irritants or tissue damage. This response is usually mediated by immune cells [1]. B cell activation factor (BAFF), a vital homeostatic cytokine, enhances B cell survival and regulates innate immune responses [2]. It is a type II membrane protein, produced and released by myeloid cells. It is expressed in a soluble form or on the cell surface [3]. Its receptors include (i) transmembrane activator, calcium modulator and cyclophilin ligand interactor (TACI), (ii) B cell maturation antigen (BCMA), and (iii) BAFF receptor (BAFF-R). The interaction between BAFF and BAFF-R is strong, unique, and highly selective [4].

Tumor necrosis factor-α (TNF-α) is an inflammatory cytokine with a wide spectrum of biological activity, and BAFF is a member of the TNF-α family [5]. As a proinflammatory cytokine, BAFF is elevated in patients with autoimmune or inflammatory diseases, such as inflammatory bowel disease, periodontitis, systemic sclerosis and so on [6–9]. Moreover, there is some research showing the expression of BAFF in reproductive tissues or female serum as well as addressing the importance of this cytokine in different reproductive diseases [10, 11]. During the early pregnancy, BAFF was rich in decidua and trophoblast. It was decreased in the patients with recurrent spontaneous miscarriage. BAFF might guide the maternal leukocytes to keep away from adverse immune responses and play a potentially vital role for successful pregnancy. During the first trimester of pregnancy, serum BAFF could serve as a predictor of hypertensive disorders that are a leading mortality cause.

However, whether the BAFF gene induces inflammation in the human fallopian tube leading to tubal pregnancy is presently unknown. Our previous study firstly showed that BAFF immunostaining appeared on the cellular membrane and in the cytoplasm of tubal epithelial cells. Both BAFF protein and mRNA in inflamed fallopian tubes had higher expression levels than those in normal fallopian tubes [12]. The present study was carried out to investigate the expression of BAFF mRNA and protein in human normal, salpingitis and tubal pregnancy tissues. Furthermore, serum levels of BAFF, TNF-α and interleukin (IL)-6 were measured using enzyme linked immunosorbent assay (ELISA) kits.

## Methods

### Patients and samples

Seventy patients undergoing salpingectomy for salpingitis (*n* = 35) and tubal pregnancy (*n* = 35) were recruited into salpingitis group and tubal pregnancy group, respectively. Twenty patients with benign uterine diseases undergoing complete hysterectomy and salpingectomy were recruited into control group. The information about the clinical characteristics of the enrolled patients was reported in Table 1. There was no significant difference between the three groups. The diagnosis and inclusion criteria in the three groups were showed separately in Table 2. The exclusion criteria for all patients were: ① using exogenous hormone or pharmacologic treatment within the last 3 months before surgery; ② having comorbidities, such as hypertension, diabetes, tuberculosis, tumors or disease of immune system; or ③ combined gynecological disorders, such as endometriosis, polycystic ovarian syndrome (PCOS) or gynecological tumors. This study has been performed in accordance with the Declaration of Helsinki and approved by the Ethics Committee of Zhuhai Municipal Maternal and Children’s Health Hospital. Written informed consents from all patients were obtained prior to the study.

**Table 1:** Characteristics of patients compared between the three groups (expressed as mean ± SD or percentage)

Characteristics	Control group (*n* = 20)	Salpingitis group (*n* = 35)	Tubal pregnancy group (*n* = 35)
Age (year)	36.55 ± 3.68	36.09 ± 3.72	34.49 ± 4.02
Body Mass Index (BMI)	23.28 ± 3.30	22.82 ± 3.16	22.86 ± 3.94
Previous abdominal/pelvic surgery (%)	35.0%	37.1%	42.9%

Note: No difference between the three groups (*P*>0.05). Previous abdominal/pelvic surgery including appendectomy, cesarean section or previous tubal pregnancy, etc.

**Table 2:** Diagnosis and inclusion Criteria in the three groups

group	diagnosis and inclusion Criteria
Salpingitis group	① HSG diagnosis of salpingitis or fallopian tube occlusion or hydrosalpinx
	② History of pelvic inflammatory disease or previous tubal pregnancy
	③ Pathological diagnosis of inflammatory changes in the epithelium of the fallopian tube
Tubal pregnancy group	① Spontaneous conception with no history of assisted reproduction
	② Diagnosis of tubal pregnancy in the ampullary region
	③ No intrauterine device and receive methotrexate treatment before surgery
	④ No acute hemorrhagic shock
Control group	① Ultrasound diagnosis of hysteromyoma and/or patients’ serious symptoms with surgical indications
	② Endometrial hyperplasia but no reproductive requirement
	③ CINIII combined with other benign gynecological disorders but no reproductive requirement

Seventy-two patients were treated with laparoscopic surgery under general anesthesia. Panoramic laparoscopic pelvic view showed the uterus, fallopian tubes, ovaries, and uterovesical. Eighteen patients underwent abdominal hysterectomy and salpingectomy under continuous epidural anesthesia. The patients in salpingitis group and tubal pregnancy group were suffered from mono/bilateral salpingectomy, and the patients in control group underwent hysterectomy and bilateral salpingectomy excluding mono/bilateral oophorectomy. The tissue samples of fallopian tube were collected during the surgical operation, immediately frozen in liquid nitrogen and subsequently stored at − 70 °C until further process for quantitative real-time polymerase chain reaction (qPCR) and Western blotting. Peripheral blood samples from all patients were collected in the morning (between 8 am and 10 am) in fasting status. All samples were allowed to clot for 2 h at room temperature before centrifugation for 20 min at approximately 1000×g. Aliquots of serum from each sample were collected and stored at − 70 °C until analyzed for ELISA.

### qPCR

Total RNA was isolated from tissue samples using the TRIzol reagent (Roche, Basel, Switzerland) according to the manufacturer’s instructions, and complementary DNA was synthesized using superscript II (Invitrogen, Carlsbad, CA, USA). The forward and reverse primers of BAFF gene were 5′-CTGATAAGACCTACGCCAT-3′ and 5′-GCTACAGACATGGTGTAAGT-3′. A 40 μl reaction mixture containing 2 × SYBR Green PCR Master Mix (Toyobo, Osaka, Japan), and forward and reverse primers is performed. This was followed by 40 cycles of denaturation at 95 °C for 15 s, annealing at 60 °C for 15 s and extension at 72 °C for 30 s after conducted at 50 °C for 2 min and a 10-min incubation at 95 °C. The data were collected and analyzed using the ABI PRISM 7300 sequence detection system (ABI, Foster City, CA, USA). The relative quantitation method was used to estimate the levels of messages encoded by this gene in each sample, with 18S ribosome mRNA serving as the endogenous normalization control. The data from the qPCR was converted to 2^-Ct^, where Ct represents the threshold cycle. The mean Ct value of the triplicate PCRs was determined, and the mean 2^-∆∆Ct^ was calculated from the triplicate cDNAs [13].

### Western blotting

Cell lysis buffer and protease inhibitor cocktail were used to lyse the tissue samples on ice, and then the supernatants were collected as the total cell lysates. Protein concentrations were measured using the bicinchoninic acid protein assay kit (Pierce, Rockford, USA). Total proteins from each sample were denatured in Laemmli buffer, fractionated by 10% SDS-PAGE, and transferred to nitrocellulose membrane. The membrane was blocked in 5% non-fat milk in TBST (Tris buffered saline plus 0.1% Tween20) for 1 h, and then incubated with antibodies against human BAFF (1:1000, Abcam, England) overnight at 4 °C. Endogenous β-actin expression served as an internal control. The immunoreactive protein complexes were detected using an enhanced chemiluminescence detection system (ECL, Beyotime, Shanghai, China) according to the manufacturer’s protocol. Band intensities were quantified by scanning densitometry by means of the Bio-Rad Quantity One software (Bio-Rad Laboratories, CA, USA).

### Elisa

Serum levels of BAFF, TNF-α and IL-6 were measured by ELISA using kits purchased from Biomatik (Canada). Briefly, 96-well microtiter plates pre-coated with BAFF, TNF-α and IL-6 antibody were incubated with suitable dilutions of the serum. Bound cytokines were detected using enzyme-linked detection antibodies as per the manufacturer’s protocol. The optical density (OD) in the wells was measured at 450 nm, and background values were subtracted. The concentration of BAFF, TNF-α and IL-6 in the samples was then determined by comparing the OD of the samples to the standard curve. The average of duplicate measurements was taken.

### Statistical analysis

Statistical Package of Social Science Software program (SPSS, Chicago, IL, USA), Windows version 20.0 was used to analyze data. Data were presented as the mean ± SD or percentage. One-way ANOVA and Chi-square tests were used. The number of subjects in this study was calculated assuming *α* = 0.05. *P* values less than 0.05 were considered significant.

## Results

**Fig. 1:**
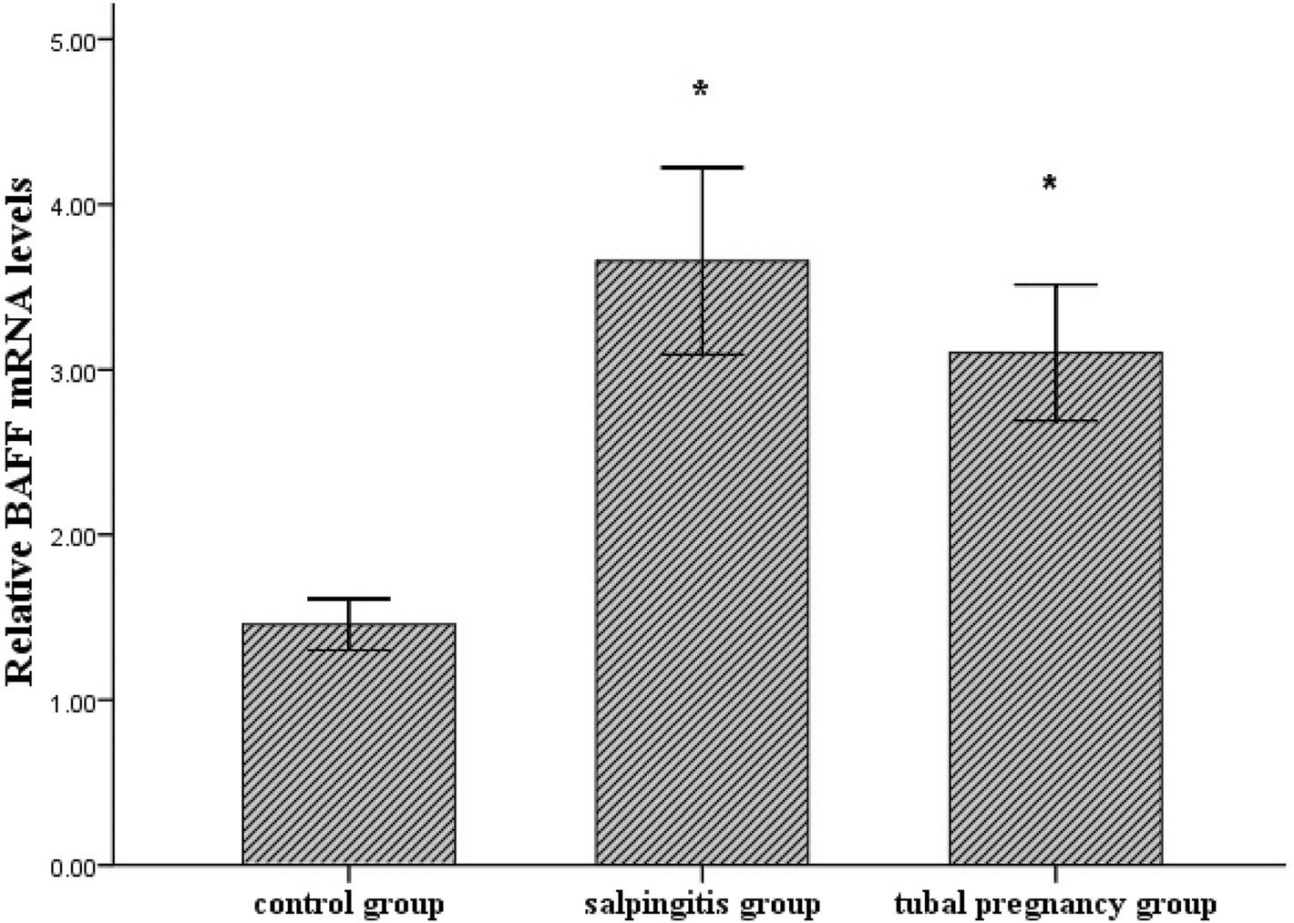
Relative BAFF mRNA levels were analyzed using qPCR. Results were shown as the mean ± SD. *P < 0.01, salpingitis group (*n* = 35) or tubal pregnancy group (*n* = 35) vs. control group (*n* = 20). *P*>0.05, salpingitis group vs. tubal pregnancy group

BAFF mRNA expression levels were illustrated in Fig. 1. The expression of BAFF mRNA in salpingitis group or tubal pregnancy group was significantly increased when compared to that in control group (*P* < 0.01). There was no significant difference in the expression of BAFF mRNA between salpingitis group and tubal pregnancy group (*P*>0.05).

**Fig. 2:**
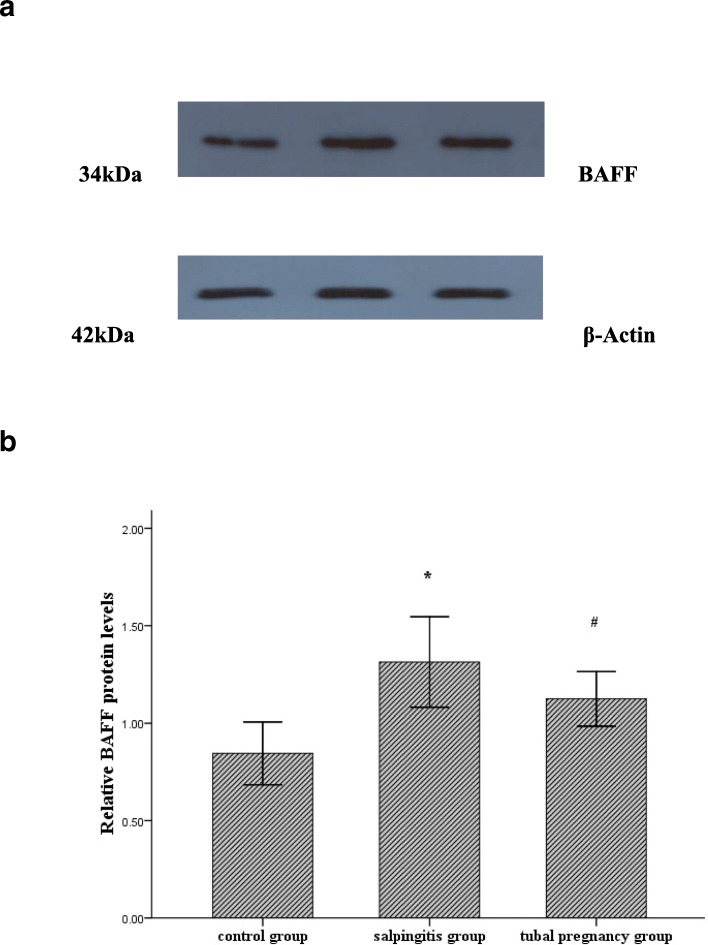
BAFF protein and β-Actin were detected as the first band and the second band, respectively. Intensity of BAFF protein was shown in control group (lane 1), salpingitis group (lane 2) and tubal pregnancy group (lane 3) (**a**). Relative BAFF protein levels were analyzed, and results were shown as the mean ± SD. *P < 0.01, salpingitis group (*n* = 35) vs. control group (*n* = 20). #*P* < 0.05, tubal pregnancy group (*n* = 35) vs. control group (*n* = 20). *P*>0.05, salpingitis group vs. tubal pregnancy group (**b**)

BAFF protein and β-Actin bands were revealed by Western blotting analysis in control group, salpingitis group and tubal pregnancy group (Fig. 2a). Intensities of BAFF protein in control group was lower than that in salpingitis group (*P* < 0.01) or in tubal pregnancy group (*P* < 0.05). There was no significant difference in BAFF protein level between salpingitis group and tubal pregnancy group (*P*>0.05) (Fig. 2b).

Serum levels of BAFF, TNF-α and IL-6 (expressed as mean ± SD) were compared between the three groups. Serum levels of BAFF, TNF-α and IL-6 were significantly higher in salpingitis group or in tubal pregnancy group when compared to control group (*P* < 0.01) (Table 3).

**Table 3:** Comparison of serum levels of BAFF, TNF-α and IL-6 (expressed as mean ± SD) between the three groups

	Control group (*n* = 20)	Salpingitis group (*n* = 35)	Tubal pregnancy group (*n* = 35)
BAFF (pg/lm)	18.05 ± 6.94^*^	37.02 ± 13.81	36.47 ± 14.81
TNF-α (pg/lm)	11.84 ± 3.11^*^	27.36 ± 11.26	32.03 ± 11.56
IL-6 (pg/lm)	4.29 ± 1.83^*^	26.02 ± 9.97	22.33 ± 8.01

Note: ^*^P < 0.01, salpingitis group or tubal pregnancy group vs. control group. No difference between salpingitis group and tubal pregnancy group (*P*>0.05)

## Discussion

Tubal pregnancy is recognized as one of the most common ectopic pregnancy types [14]. It is well-known in humans, but is rarely diagnosed in animals [15]. The fallopian tubes provide the complex environment required for fertilization, pre-implantation development of the embryo, and gamete transport to the uterine cavity. Although the etiology of human tubal pregnancy remains unclear, much of the literatures show that tubal pregnancy occurs due to inflammatory factors [16–18]. Salpingitis may result in tubal pregnancy by causing fallopian tube occlusion and hydrosalpinx [19–21]. Moreover, the inflammatory environment within the fallopian tube may disrupt ciliary beat activity and smooth muscle contractility and thus affect embryo-tubal transport [22]. The pro-inflammatory cytokines amplify the inflammatory process and destruction in fallopian tube. TNF-α and IL-6 are among these cytokines [23, 24].

We found that serum TNF-α and IL-6 were expressed significantly higher in patients with salpingitis and tubal pregnancy, which supported previous studies [25, 26]. They were expressed more in women with ectopic pregnancy than normal pregnancy or miscarriage. TNF-α, together with IL-6, could predict ectopic pregnancy, with 100% of specificity, but 52.9% of sensitivity. The serum IL-6 was significantly increased in the patients with tubal ectopic pregnancy vs. normal pregnancy or intrauterine miscarriage. It has served as a biomarker for ectopic pregnancy.

As a TNF superfamily molecule, the transcript of BAFF is up-regulated by TNF-α [27]. Interaction between TNF-α-induced BAFF and BAFF-mediated VEGF may prevent the B cells from apoptosis, or maintain the supply of oxygen and nutrients in inflammatory microenvironments [28]. Increased BAFF may lead to B cell provocation, and raise activation of T cells or dendritic cells (DC) in the overall inflammatory burden. BAFF also helps DC maturation and IL-6 release [29]. IL-6 has an inhibitory effect on ciliary activity [30]. In this study, serum levels of BAFF, TNF-α and IL-6 were all significantly increased in patients with salpingitis and tubal pregnancy in comparison to control group. Moreover, the BAFF mRNA and protein expression levels in tissue samples from patients with salpingitis and tubal pregnancy were higher than those in control group. Increased levels of BAFF might change the microenvironment for fertilization and inflammatory responses on human fallopian tube.

Epidemiological studies show that pro-inflammatory factors act a potential role in multiple diseases. The expression of pro-inflammatory factors, such as IL-1, IL-6, TNF-α and COX-2, dues to the histological heterogeneity of ovarian cancers [31]. Inflammation is an essential process in the pathogenesis of endometriosis. There is some research showing that the expression of BAFF, TNF-α or IL-6 may be affected in case of endometriosis [32, 33]. BAFF protein was found elevated in the serum of endometriosis patients. TNF-α or IL-6 levels in serum and peritoneal fluid samples played a role in endometriosis-related pelvic inflammation. Moreover, some studies report that BAFF is produced by adipocytes and mediates metabolic syndrome in obesity as an autocrine and/or paracrine factor [34, 35]. In our study, we had excluded patients who were suffered from endometriosis, diabetes, hypertension, PCOS, tuberculosis, disease of immune system or tumors in order to avoid some potential bias.

Several limitations of this study warrant mention. First, The tissue and peripheral blood samples were collected from patients in our study. There are some confounders, such as gynecological disorders, diabetes, hypertension, tumors or pharmacologic treatments, that may have significantly affected the results. We had the greatest exclusion of these confounders, but the results might be biased due to unmeasured confounders. Second, the enrolled women, as well as demographic and clinical characteristics, might be more detailed in order to avoid possible biases. Therefore, we had reported detailed information of the selection of patients, such as age, BMI or previous abdominal/pelvic surgery. Third, tubal pregnancy is well-known in humans, but is rarely diagnosed in animals. It will be a great challenge to study tubal pregnancy using animal models. If it is possible, the results might be confirmed by animal experiments in the future.

## Conclusions

To our knowledge, this is the first study to evaluate the association of human tubal pregnancy and BAFF gene. We found significantly increased expressions of BAFF mRNA or protein in whole tissue samples, and serum levels of BAFF, TNF-α and IL-6 in whole blood samples from patients with salpingitis and tubal pregnancy, in comparison to the control group. Based on the obtained results, high expression of BAFF gene might induce inflammation in the human fallopian tube, suggesting a possible role in the tubal pregnancy process. Further research needs to study the BAFF gene for the advancement of improved preventative measures, diagnostic screening methods and novel treatments for human tubal pregnancy.

## Abbreviations

BAFF: B cell activation factor
BAFF-R: B cell activation factor-receptor
BCMA: B cell maturation antigen
BMI: Body mass index
CIN: Cervical intraepithelial neoplasia
COX-2: Cyclooxygenase-2
DC: Dendritic cells
ELISA: Enzyme linked immunosorbent assay
HSG: Hysterosalpingography
IL: Interleukin
PCOS: Polycystic ovarian syndrome
qPCR: Quantitative real-time polymerase chain reaction
TACI: Transmembrane activator, calcium modulator and cyclophilin ligand interactor
TNF-α: Tumor necrosis factor-α

## References

[1] Stutz A, Golenbock DT, Latz E (2009). Inflammasomes: too big to miss. J Clin Invest.

[2] Thompson JS, Bixler SA, Qian F, Vora K, Scott ML, Cachero TG (2001). BAFF-R, a newly identified TNF receptor that specifically interacts with BAFF. Science.

[3] Nardelli B, Belvedere O, Roschke V, Moore PA, Olsen HS, Migone TS (2001). Synthesis and release of B-lymphocyte stimulator from myeloid cells. Blood.

[4] Bossen C, Schneider P (2006). BAFF, APRIL and their receptors: structure, function and signaling. Semin Immunol.

[5] Bolkun L, Lemancewicz D, Jablonska E, Kulczynska A, Bolkun-Skornicka U, Kloczko J (2014). BAFF and APRIL as TNF superfamily molecules and angiogenesis parallel progression of human multiple myeloma. Ann Hematol.

[6] Koizumi M, Hiasa Y, Kumagi T, Yamanishi H, Azemoto N, Kobata T (2013). Increased B cell-activating factor promotes tumor invasion and metastasis in human pancreatic cancer. PLoS One.

[7] Uzzan M, Colombel JF, Cerutti A, Treton X, Mehandru S (2016). B cell-activating factor (BAFF)-targeted B cell therapies in inflammatory bowel diseases. Dig Dis Sci.

[8] Gümüş P, Nizam N, Lappin DF, Buduneli N (2014). Saliva and serum levels of B-cell activating factors and tumor necrosis factor-α in patients with periodontitis. J Periodontol.

[9] Sanges S, Guerrier T, Launay D, Lefèvre G, Labalette M, Forestier A (2017). Role of B cells in the pathogenesis of systemic sclerosis. Rev Med Interne.

[10] Guo WJ, Qu X, Yang MX, Zhang WD, Liang L, Shao QQ, Kong BH (2008). Expression of BAFF in the trophoblast and decidua of normal early pregnant women and patients with recurrent spontaneous miscarriage. Chin Med J.

[11] Stohl HE, Lee RH, Manetta J, Kikly K, Korst LM, Stohl W (2017). Maternal serum B-cell activating factor levels: candidate early biomarker for hypertensive disorders of pregnancy. Hypertension.

[12] Xu JY, Yang GY, Kuang GC, Huang JL (2014). Expression and change of B cell activation factor of the TNF family (BAFF) gene between human Normal and inflamed fallopian tubes. J Reprod Contracept.

[13] Juhasz A, Frankel P, Cheng C, Rivera H, Vishwanath R, Chiu A (2003). Quantification of chemotherapeutic target gene mRNA expression in human breast cancer biopsies: comparison of real-time reverse transcription-PCR vs. relative quantification reverse transcription-PCR utilizing DNA sequencer analysis of PCR products. J Clin Lab Anal.

[14] Corpa JM (2006). Ectopic pregnancy in animals and humans. Reproduction.

[15] Schlabritz-Loutsevitch NE, Hubbard GB, Frost PA, Cummins LB, Dick EJ, Nathanielsz PW (2004). Abdominal pregnancy in a baboon: a first case report. J Med Primatol.

[16] Shao R, Feng Y, Zou S, Li X, Cui P, Billig H (2015). Quantitative analysis of hormones and inflammatory cytokines in chlamydia trachomatis-infected women with tubal ectopic pregnancy and early intrauterine pregnancy. Data Brief.

[17] Rausch ME, Barnhart KT (2012). Serum biomarkers for detecting ectopic pregnancy. Clin Obstet Gynecol.

[18] Huang HY, Chan SH, Wu CH, Wang CW, Lai CH, Soong YK (2005). Interleukin-1 system messenger ribonucleic acid and protein expression in human fallopian tube may be associated with ectopic pregnancy. Fertil Steril.

[19] Islam A, Fawad A, Shah AA, Jadoon H, Sarwar I, Abbasi AU (2017). Analysis of two years cases of ectopic pregnancy. J Ayub Med Coll Abbottabad.

[20] Chu J, Harb HM, Gallos ID, Dhillon R, Al-Rshoud FM, Robinson L (2015). Salpingostomy in the treatment of hydrosalpinx: a systematic review and meta-analysis. Hum Reprod.

[21] Shao R, Zhang SX, Weijdegård B, Zou S, Egecioglu E, Norström A (2010). Nitric oxide synthases and tubal ectopic pregnancies induced by chlamydia infection: basic and clinical insights. Mol Hum Reprod.

[22] Shaw JL, Dey SK, Critchley HO, Horne AW (2010). Current knowledge of the aetiology of human tubal ectopic pregnancy. Hum Reprod Update.

[23] Luo HJ, Xiao XM, Zhou J, Wei W (2015). Therapeutic influence of intraperitoneal injection of Wharton's jelly-derived mesenchymal stem cells on oviduct function and fertility in rats with acute and chronic salpingitis. Genet Mol Res.

[24] Yousefian E, Novin MG, Fathabadi FF, Farahani RM, Kachouei EY (2013). The expression of IL-6Rα and Gp130 in fallopian tubes bearing an ectopic pregnancy. Anat Cell Biol.

[25] Soriano D, Hugol D, Quang NT, Darai E (2003). Serum concentrations of interleukin-2R (IL-2R), IL-6, IL-8, and tumor necrosis factor alpha in patients with ectopic pregnancy. Fertil Steril.

[26] Rajendiran S, Senthil Kumar GP, Nimesh A, Dhiman P, Shivaraman K, Soundararaghavan S (2016). Diagnostic significance of IL-6 and IL-8 in tubal ectopic pregnancy. J Obstet Gynaecol.

[27] Alsaleh G, Messer L, Semaan N, Boulanger N, Gottenberg JE, Sibilia J (2007). BAFF synthesis by rheumatoid synoviocytes is positively controlled by alpha5beta1 integrin stimulation and is negatively regulated by tumor necrosis factor alpha and toll-like receptor ligands. Arthritis Rheum.

[28] Lee GH, Lee J, Lee JW, Choi WS, Moon EY (2013). B cell activating factor-dependent expression of vascular endothelial growth factor in MH7A human synoviocytes stimulated with tumor necrosis factor-α. Int Immunopharmacol.

[29] Moisini I, Davidson A (2009). BAFF: a local and systemic target in autoimmune diseases. Clin Exp Immunol.

[30] Papathanasiou A, Djahanbakhch O, Saridogan E, Lyons RA (2008). The effect of interleukin-6 on ciliary beat frequency in the human fallopian tube. Fertil Steril.

[31] Plewka D, Kowalczyk AE, Jakubiec-Bartnik B, Morek M, Bogunia E, Kmiec A (2014). Immunohistochemical visualization of pro-inflammatory cytokines and enzymes in ovarian tumors. Folia Histochem Cytobiol.

[32] Jin CH, Yi KW, Ha YR, Jin CH, Yi KW, Ha YR (2015). Chemerin expression in the peritoneal fluid, serum, and ovarian Endometrioma of women with endometriosis. Am J Reprod Immunol.

[33] Hever A, Roth RB, Hevezi P, Marin ME, Acosta JA, Acosta H (2007). Human endometriosis is associated with plasma cells and overexpression of B lymphocyte stimulator. Proc Natl Acad Sci U S A.

[34] Do MS, Jeong HS, Choi BH, Hunter L, Langley S, Pazmany L (2006). Inflammatory gene expression patterns revealed by DNA microarray analysis in TNF-alpha-treated SGBS human adipocytes. Yonsei Med J.

[35] Kim DH, Do MS (2015). BAFF knockout improves systemic inflammation via regulating adipose tissue distribution in high-fat diet-induced obesity. Exp Mol Med.

